# Macrophage extracellular traps require peptidylarginine deiminase 2 and 4 and are a source of citrullinated antigens bound by rheumatoid arthritis autoantibodies

**DOI:** 10.3389/fimmu.2024.1167362

**Published:** 2024-02-27

**Authors:** S. Janna Bashar, Caitlyn L. Holmes, Miriam A. Shelef

**Affiliations:** ^1^ Department of Medicine, University of Wisconsin-Madison, Madison, WI, United States; ^2^ William S. Middleton Memorial Veteran’s Hospital, Madison, WI, United States

**Keywords:** macrophage extracellular trap, citrullinated antigens, anti-citrullinated protein antibodies, rheumatoid arthritis, peptidylarginine deiminase 2, peptidylarginine deiminase 4

## Abstract

**Introduction:**

Anti-citrullinated protein antibodies (ACPAs) are a hallmark of rheumatoid arthritis, but the sources of citrullinated antigens as well as which peptidylarginine deiminases (PADs) are required for their production remain incompletely defined. Here, we investigated if macrophage extracellular traps (METs) could be a source of citrullinated proteins bound by APCAs, and if their formation requires PAD2 or PAD4.

**Methods:**

Thioglycolate-induced peritoneal macrophages from wild-type, PAD2^-/-^, and PAD4^-/-^ mice or human peripheral blood-derived M1 macrophages were activated with a variety of stimulants, then fixed and stained with DAPI and either anti-citrullinated histone H4 (citH4) antibody or sera from ACPA+ or ACPA- rheumatoid arthritis subjects. METs were visualized by immunofluorescence, confirmed to be extracellular using DNase, and quantified.

**Results:**

We found that ionomycin and monosodium urate crystals reliably induced murine citH4+ METs, which were reduced in the absence of PAD2 and lost in the absence of PAD4. Also, IgG from ACPA+, but not ACPA-, rheumatoid arthritis sera bound to murine METs, and in the absence of PAD2 or PAD4, ACPA-bound METs were lost. Finally, ionomycin induced human METs that are citH4+ and ACPA-bound.

**Discussion:**

Thus, METs may contribute to the pool of citrullinated antigens bound by ACPAs in a PAD2- and PAD4-dependent manner, providing new insights into the targets of immune tolerance loss in rheumatoid arthritis.

## Introduction

Citrullinated proteins, formed by the post-translational modification of arginines to citrullines, are targeted by anti-citrullinated protein antibodies (ACPAs) in ~75% of people with rheumatoid arthritis (RA), a chronic autoimmune disease (1). There are five isoforms of human peptidylarginine deiminases (PADs) that catalyze citrullination, but primarily PAD2 and PAD4 have been implicated in RA due to their presence in immune cells (2) and RA synovial tissue (3), their contributions to murine inflammatory arthritis (4, 5), and the presence of RA-associated single nucleotide polymorphisms in both *PADI2* and *PADI4* (6, 7). However, the mechanisms by which these two PADs contribute to the high levels of citrullinated proteins in RA (8) that are bound by APCAs remain incompletely defined.

PAD4 is required for histone citrullination and the formation of citrullinated neutrophil extracellular traps (NETs) (9, 10), extracellular structures composed of decondensed chromatin and cellular proteins that are important in an antimicrobial response but also increased in RA and bound by ACPAs (11–13). Thus PAD4, via NETs, may provide citrullinated antigens, driving autoimmune responses in RA. However, despite being required for the formation of citrullinated NETs, PAD4 was not required for increased citrullination in murine inflammatory arthritis (4, 5). In contrast, PAD2 was required for joint citrullination in murine inflammatory arthritis but not NET production (5, 10). Thus, there may be additional sources of citrullinated antigens beyond NETs.

Macrophages also produce extracellular traps (METs) (14–16), but their role in RA is only emerging. RA synovial macrophage numbers correlate with increased articular destruction (17), while depletion reduces arthritis severity, splenic citrullination, and anti-cyclic citrullinated peptide (CCP) titers in murine inflammatory arthritis (18, 19). Further, citrullinated proteins, PAD4, and PAD2 appear to colocalize with macrophages in inflammatory tissue, and activated murine and RA synovial macrophages generate citrullinated METs (19, 20). However, it remains unknown if PAD4 or PAD2 is required for MET formation and if METs can make citrullinated proteins bound by ACPAs.

In this study we investigate the requirement of PAD4 and PAD2 in the production of METs that could be a source of citrullinated proteins bound by ACPAs.

## Materials and methods

### Mice

Similar numbers of male and female age- and sex-matched adult wild-type, PAD4^-/-^ (9, 21) and PAD2^-/-^ (22) mice back-crossed 12 generations to the C57BL/6 background were used. Animals were housed in a specific pathogen-free facility and experiments were approved by the University of Wisconsin Institutional Animal Care and Use Committee.

### Human subjects

Human subjects research was approved by the University of Wisconsin Institutional Review Board and complied with the Helsinki Declaration. Sera from five RA subjects with anti–CCP and rheumatoid factor levels >2x the upper limit of normal were combined to generate the ACPA+ serum pool. Sera from five RA subjects with negative anti-CCP and rheumatoid factor test results were combined for the ACPA- serum pool. Samples were obtained from the University of Wisconsin Rheumatology Biorepository (23).

### Murine macrophage isolation

Four days after intraperitoneal injection with aged 4% thioglycolate (Hardy Diagnostics), mice were euthanized for peritoneal lavage with 6-7 ml of cold RPMI with 2% heat-inactivated fetal bovine serum (FBS). The peritoneal fluid was centrifuged at 500xg for 5 minutes and the cell pellet resuspended in RPMI with L-glutamine, 2% FBS, and penicillin-streptomycin (macrophage media). The cell suspension was incubated in non-tissue culture treated plastic dishes at 37°C with 5% CO_2_ in a humidified incubator. The next day, the supernatant was aspirated, and the wells washed with RPMI and then phosphate buffered saline (PBS) prior to incubation with cold 2mM EDTA on ice for 30 minutes. Detached cells were collected, centrifuged at 500xg for 5 minutes, and resuspended in fresh macrophage media. By flow cytometry, cells were on average 86% F4/80^+^ macrophages and 0.17% Ly6G^+^Ly6C^+^ neutrophils with the remainder of cells primarily B cells and monocytes.

### Human peripheral blood-derived M1 macrophage preparation

Frozen human peripheral blood-derived M1 macrophages (polarized with lipopolysaccharide [LPS] and interferon-gamma) from three independent healthy donors (StemCell Technologies) were thawed at 37°C until a small frozen cell pellet remained. The cell suspension was gently brought to a final volume of 20 ml of 37°C RPMI with 10% FBS, followed by centrifugation at 300xg for 10 minutes, gentle resuspension with 15 ml of 10% FBS in RPMI, and repeat centrifugation at 300xg for 10 minutes. Cells were resuspended in macrophage media and confirmed to be 99% CD14^+^CD80^+^CD68^+^ macrophages with no CD11b^+^CD15^+^CD14^-^ neutrophils by flow cytometry.

### Macrophage stimulation

Murine macrophages (3-6x10^4^ cells/ml) were seeded in 500 µl of macrophage media in 24-well tissue culture plates containing acid washed glass coverslips, followed by incubation at 37°C with 5% CO_2_ for 30-60 minutes. After optimization of stimulant doses, cells received no treatment or were treated with 5x10^6^ heat-killed *Candida albicans* (InvivoGen), 20 μM ionomycin (MilliporeSigma), 30 μg/mL LPS from E. coli (InvivoGen), 1.2 mg/mL monosodium urate (MSU) crystals (InvivoGen), 10 μg/mL platelet activating factor (PAF, MilliporeSigma), 50 nM phorbol myristate acetate (PMA, MP Biomedicals), or 400 ng/mL tumor necrosis factor alpha (TNFα, R&D Systems) followed by incubation for 20 hours at 37°C with 5% CO_2_.

Human macrophages (8x10^4^ cells/ml) were seeded as above and incubated at 37°C with 5% CO_2_ for 4 hours. After optimization of stimulant doses, cells received no treatment or were treated with 6.5 μM ionomycin (MP Biomedicals) or 25 ng/mL TNFα (R&D Systems) followed by the above incubation parameters. The following concentrations of MSU were tested: 1.2 mg/ml, 400 µg/ml, and 130 µg/ml.

### DNase experiments

After incubation with stimulants for 20 hours, coverslips were washed with PBS and one coverslip from each condition (per experiment) was incubated with 20 U/ml of DNase I in 1x DNase buffer (New England Biolabs) in PBS at 37°C with 5% CO_2_ for 20 minutes, while paired coverslips were incubated with DNase buffer in PBS without DNase I.

### Immunofluorescence

Cells were prepared for immunofluorescence as previously described (10). For histone citrullination experiments, coverslips were washed with PBS, then treated with a solution of 4% paraformaldehyde, 1% NP-40, and 0.5% Triton X-100 in PBS for 30 minutes at 4°C, washed, blocked for 1 hour at room temperature with antibody blocking solution (2.5% bovine serum albumin, 5% goat serum, and 0.5% Tween-20 in PBS), incubated overnight at 4°C in a humidified chamber with rabbit anti-mouse histone H4 (citrulline 3) IgG (MilliporeSigma) diluted 1:100 in antibody blocking solution, washed, and incubated for 1 hour at room temperature with a 1:200 dilution of donkey anti-rabbit IgG conjugated to TRITC (Jackson Laboratories) and a 1:1000 dilution of 4′,6-diamidino-2-phenylindole (DAPI) in antibody blocking solution. Coverslips were washed and mounted on glass microscope slides using Prolong Diamond Antifade (Molecular Probes).

For ACPA binding experiments, coverslips were fixed with 4% paraformaldehyde in PBS for 30 minutes at 4°C, washed, and then blocked in either murine ACPA blocking solution (2.5% bovine serum albumin and 5% goat serum in PBS) for 3 hours at 4°C or human ACPA blocking solution (5% bovine serum albumin and 10% goat serum) with human IgG1 Fc block (BD Biosciences) in PBS overnight at 4°C, before incubation with ACPA+ or ACPA- sera diluted 1:75 in respective blocking solution overnight at 4°C in a humidified chamber. Coverslips were then washed, permeabilized with 1% NP-40, 0.5% Triton X-100, and 0.5% Tween-20 in PBS for 30 minutes at 4°C, washed, incubated with 1:200 goat IgG anti-human IgG conjugated to TRITC (SouthernBiotech) and 1:1000 DAPI in respective blocking solution with 0.5% Tween-20 for 1 hour at room temperature followed by washing and mounting as above.

### Quantification of METs

For murine experiments, a Leica fluorescence microscope with Image Pro-Plus v.6.3 (Media Cybernetics) was used to image five pre-determined areas on each coverslip at 400x. For human experiments, images were acquired on a Leica DMi8 inverted microscope with Leica Application Suite X at 200X. For each image, total cells, MET-like structures (defined as decondensed or diffuse DNA structures), and non-METs (defined as condensed nuclei) were counted by eye in a blinded manner (DNase treatment, stimulant, genotype, and ACPA presence unknown to counters) using ImageJ.

### Confocal imaging

Confocal images of coverslips were acquired with a Nikon A1-RS scanning confocal microscope with a 40x oil objective. Images were prepared with NIS-Elements software.

### Statistical analysis

Data were analyzed by paired t-test using Prism (GraphPad) with p<0.05 considered significant.

## Results

**Figure 1:**
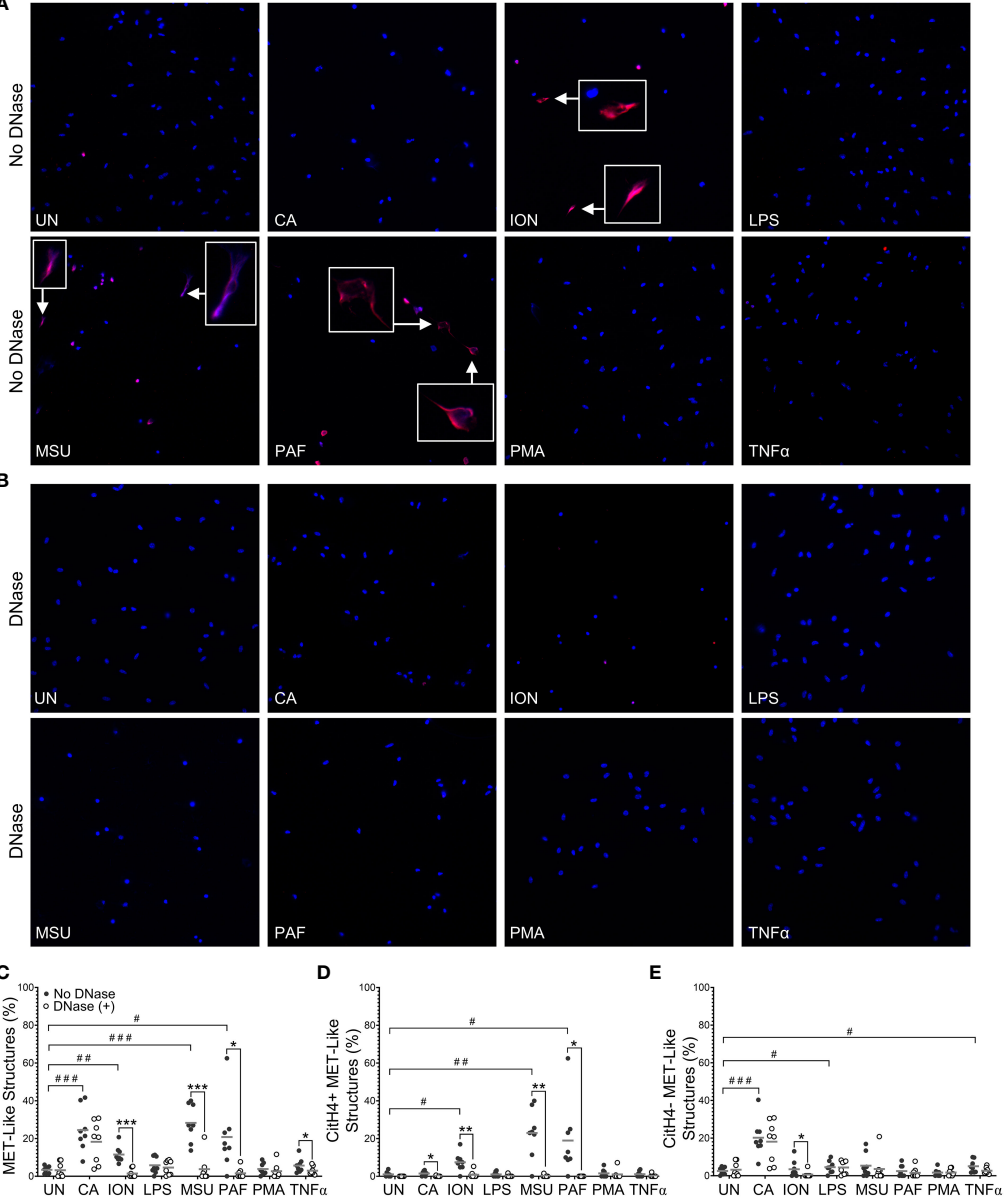
Murine macrophages produce METs in response to specific stimuli. Murine peritoneal macrophages were left untreated (UN) or were treated with heat-killed *Candida albicans* (CA), ionomycin (ION), lipopolysaccharide (LPS), monosodium urate (MSU) crystals, platelet activating factor (PAF), phorbol myristate acetate (PMA), or tumor necrosis factor alpha (TNFα) followed by treatment with no DNase or DNase, then fixation and permeabilization, staining with DAPI (blue) and anti-citrullinated histone H4 (citH4) antibody (pink), imaging, and quantification of MET-like structures. Representative images at 400x of no DNase **(A)** or DNase **(B)** treated macrophages. Arrows indicate citH4+ MET-like structures and insets are enlarged 3x. Graphs depict the means and individual values for the percent of macrophages that formed total **(C)**, citH4+ **(D)**, or citH4- **(E)** MET-like structures for each stimulant compared to untreated for the non-DNase treated samples (#p<0.05, ##p<0.01, ###p<0.001) and for DNase compared to no DNase for each stimulant (*p<0.05, **p<0.01, ***p<0.001) by paired t-test (n=8).

To evaluate stimuli that induce METs and engage PADs to produce citrullinated METs in mice, we treated murine peritoneal macrophages *in vitro* with seven stimuli previously shown to induce extracellular traps in neutrophils and/or macrophages (5, 10, 16, 19, 24). After fixation and permeabilization, cells were stained with DAPI and anti-citrullinated histone H4 (citH4, **Figure 1A**), and the percentage of cells that formed citH4+ or citH4- MET-like structures (defined by decondensed or diffuse chromatin with or without citrullinated histones) was quantified. To ensure that the MET-like structures were extracellular, cells were treated with DNase prior to permeabilization (**Figure 1B**) and MET percentages compared with non-DNase treated cells. As quantified in **Figure 1C**, *Candida*, ionomycin, MSU, and PAF induced MET-like structures, but only the MET-like structures induced by ionomycin, MSU, and PAF were reduced with DNase treatment, indicating true extracellular structures. Further, only ionomycin, MSU, and PAF significantly induced citH4+ MET-like structures, which were lost with DNase treatment (**Figure 1D**). Interestingly, *Candida*, LPS, and TNFα appeared to induce citH4- MET-like structures (**Figure 1E**). However, the MET-like structures induced by *Candida* and LPS were not reduced with DNase treatment, suggesting that they were not truly METs. Finally, although the percentage of MET-like structures was small, combining the data from **Figures 1C, E**, TNFα appeared to induce citH4- MET-like structures that were degraded by DNase and thus would be citH4- METs. Of note, no difference was observed in METs between male and female mice (**Supplementary Figure 1**). Together, these data suggest that ionomycin, MSU, and PAF are activators of DNase-accessible, true citH4+ METs in murine peritoneal macrophages. Thus, moving forwards, we use the term “MET” and not “MET-like structure”.

**Figure 2:**
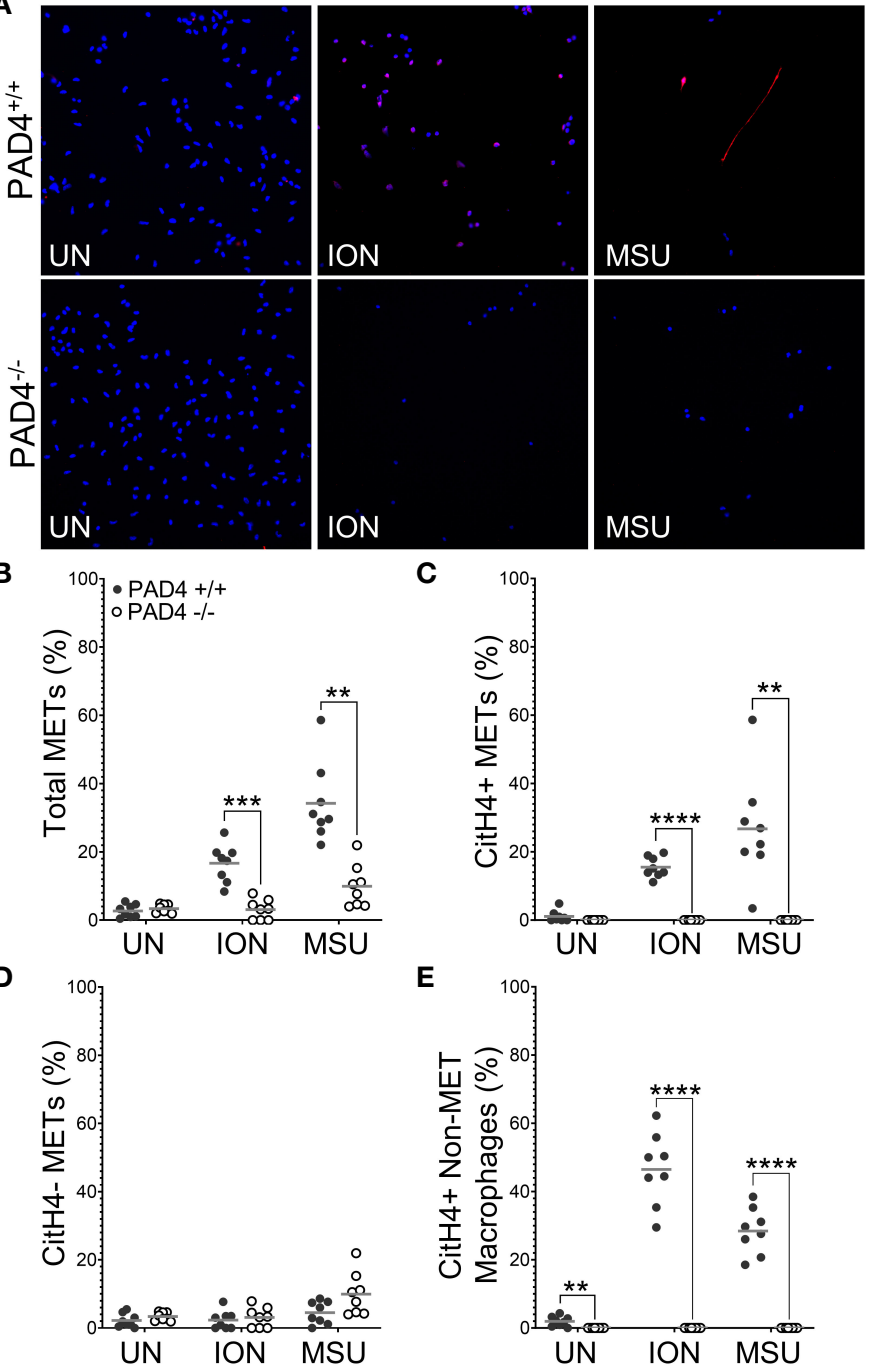
Citrullinated histone-containing murine METs and non-MET macrophages are lost in the absence of PAD4. Peritoneal macrophages from PAD4^+/+^ and PAD4^-/-^ mice were left untreated (UN) or were treated with ionomycin (ION) or monosodium urate (MSU) crystals and stained with DAPI (blue) and anti-citrullinated histone H4 (citH4) antibody (pink). **(A)** Representative images at 400x. Graphs depict the means and individual values for the percentage of macrophages that were total METs **(B)**, citH4+ METs **(C)**, citH4- METs **(D)**, and citH4+ non-MET macrophages **(E)** for each condition. For all panels: PAD4^+/+^ and PAD4^-/-^ were compared by paired t-test (**p<0.01, ***p<0.001, ****p<0.0001) and n=8.

To determine if PAD4 is required to produce citH4+ METs, we performed similar experiments as above with macrophages from PAD4^+/+^ and PAD4^-/-^ mice stimulated with ionomycin and MSU (**Figure 2**). Ionomycin mimics the calcium flux that occurs in activated immune cells, including macrophages (25), and MSU induces IL-1ß via the NLRP3 inflammasome, a pathogenic structure in RA macrophages (26, 27). Thus, both stimuli are highly relevant for RA. Because PAF-induced METosis could be variable, it was not selected for further use. As shown in **Figure 2B**, PAD4^-/-^ macrophages formed significantly fewer METs than PAD4^+/+^ macrophages in response to ionomycin and MSU. Further, PAD4^-/-^ macrophages did not produce any citH4+ METs in response to stimuli (**Figure 2C**). The small number of METs produced in the absence of PAD4 appeared to be citH4- (**Figure 2D**). In addition to quantifying citH4+ METs, we also quantified citH4+ non-MET macrophages, i.e., macrophages with condensed nuclei. CitH4+ non-MET macrophages were also nonexistent in the absence of PAD4 (**Figure 2E**). Thus, PAD4 is required for histone H4 citrullination in stimulated macrophages and for the production of citH4+ METs.

**Figure 3:**
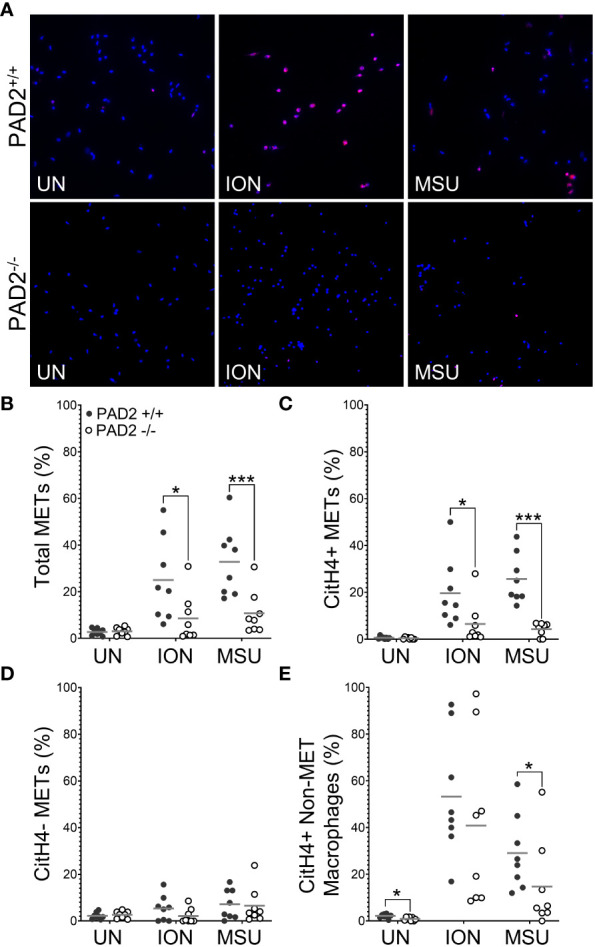
Citrullinated histone-containing murine METs are reduced in the absence of PAD2. Peritoneal macrophages from PAD2^+/+^ and PAD2^-/-^ mice were left untreated (UN) or were treated with ionomycin (ION) or monosodium urate (MSU) crystals and stained with DAPI (blue) and anti-citrullinated histone H4 (citH4) antibody (pink). **(A)** Representative images at 400x. Graphs depict the means and individual values for the percentage of total METs **(B)**, citH4+ METs **(C)**, citH4- METs **(D)**, and citH4+ non-MET macrophages **(E)** for each condition. For all panels: PAD2^+/+^ and PAD2**^-/-^** were compared by paired t-test (*p<0.05, ***p<0.001) and n=8.

We then used identical methods and PAD2^+/+^ and PAD2^-/-^ mice to determine if PAD2 is required for MET production. As shown in **Figure 3B**, total METs were reduced for PAD2^-/-^ macrophages in response to ionomycin and MSU, similar to PAD4^-/-^ macrophages. However, lack of PAD2 led to a reduction, but not complete absence, of citH4+ METs (**Figure 3C**). Similar to PAD4^-/-^ macrophages, citH4- METs were not reduced in the absence of PAD2. Finally, the number of citH4+ non-MET macrophages was only minimally reduced and only for MSU stimulation in the absence of PAD2, as opposed to a complete absence of citH4+ non-MET macrophages for the PAD4 knockout. Together these data suggest that both PAD4 and, to a lesser extent, PAD2 are required to produce citH4+ METs.

**Figure 4:**
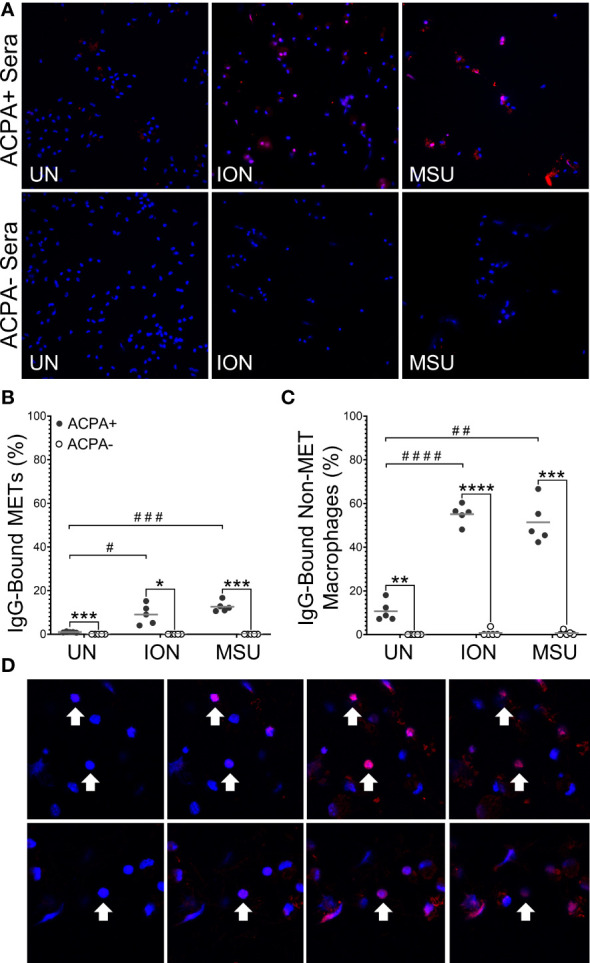
Anti-citrullinated protein antibodies (ACPAs) bind to murine METs and activated macrophages. Untreated (UN), ionomycin (ION)-treated or monosodium urate (MSU) crystal-treated macrophages were fixed, incubated with sera from ACPA+ or ACPA- rheumatoid arthritis subjects, then permeabilized and stained with DAPI (blue) and anti-human IgG antibody (pink). **(A)** Representative images at 400x. Graphs depict the means and individual values for the percentage of macrophages that were IgG-bound METs **(B)** or IgG-bound non-MET macrophages **(C)** for each stimulant compared to the untreated control for ACPA+ sera (paired t-test; #p<0.05, ##p<0.01, ###p<0.001, #### p<0.0001) and for ACPA+ versus ACPA- for each condition (paired t-test; *p<0.05, **p<0.01, ***p<0.001, ****p<0.0001); n=5. **(D)** Representative 1.5 μm z section intervals obtained by confocal microscopy of three representative non-MET macrophages (arrows) that had been incubated with ACPA+ sera.

Next, we determined if the citrullinated antigens present in murine METs or activated macrophages could be bound by ACPAs. We repeated the above experiments with wild-type mice and two modifications: (1) incubating macrophages with human sera pooled from ACPA+ or ACPA- RA subjects instead of anti-citH4 antibody and (2) permeabilization after incubation with sera, instead of before, to better mimic the endogenous biology as opposed to the goal of detecting histone citrullination in all cells. As shown in **Figures 4A, B**, essentially no binding of IgG from ACPA- RA sera to METs was detected, whereas IgG from ACPA+ RA sera bound to ionomycin- and MSU-activated METs. Binding of IgG from ACPA+ sera to METs was similar in males and females (**Supplementary Figure 2**). We also detected IgG binding for APCA+ sera, but not ACPA- RA sera, to non-MET macrophages after stimulation with ionomycin or MSU (**Figure 4C**). Since the cells were not permeabilized prior to incubation with ACPA+ sera, this observation suggested either binding to citrullinated proteins on the cell membrane or activation-induced membrane permeabilization, allowing ACPAs access to intracellular antigens. To differentiate between the two possibilities, we used confocal imaging. We observed intracellular staining with ACPA+ sera (**Figure 4D**), which is consistent with membrane permeabilization. Finally, we compared ACPA-bound METs and non-MET macrophages with and without DNase treatment (**Supplementary Figure 3**). As expected, all METs were lost upon DNase treatment. In contrast, the ACPA-bound non-MET macrophages were unaffected by DNase, suggesting that citrullinated proteins in the cytoplasm of activated macrophages are accessible to ACPAs, while the DNA in the nucleus is not accessible to DNase.

**Figure 5:**
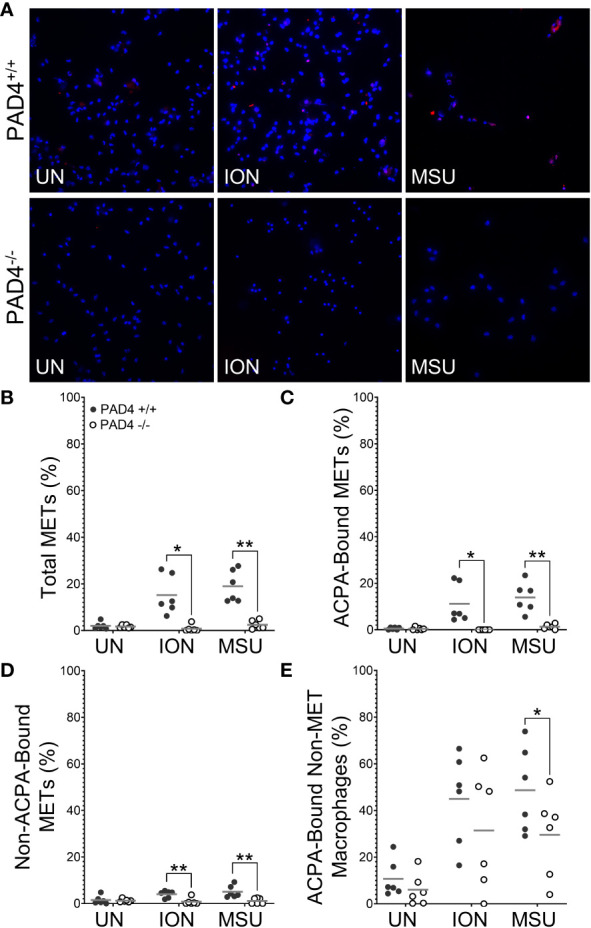
ACPA-bound murine METs are lost in the absence of PAD4. Peritoneal macrophages from PAD4^+/+^ and PAD4^-/-^ mice were left untreated (UN) or were treated with ionomycin (ION) or monosodium urate (MSU) crystals, then fixed, incubated with ACPA+ rheumatoid arthritis sera, permeabilized, and stained with DAPI (blue) and anti-human IgG antibody (pink). **(A)** Representative images at 400x. Graphs depict the means and individual values for the percentage of macrophages that are total METs **(B)**, ACPA-bound METs **(C)**, METs not bound by ACPAs **(D)**, or ACPA-bound non-MET macrophages **(E)**. For all panels: PAD4^+/+^ and PAD4^-/-^ were compared by paired t-test (*p<0.05, **p<0.01) and n=6.

**Figure 6:**
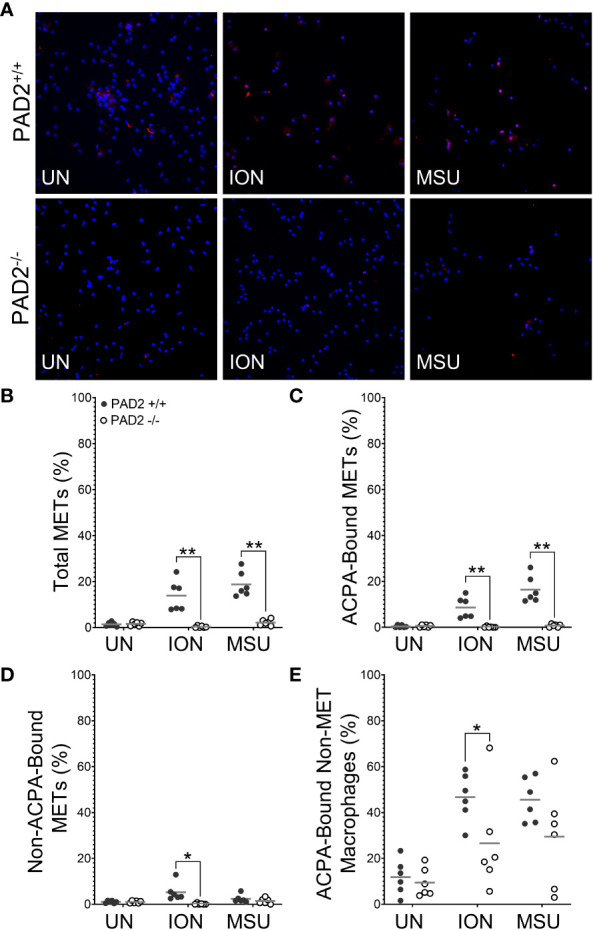
ACPA-bound murine METs are lost in the absence of PAD2. Peritoneal macrophages from PAD2^+/+^ and PAD2^-/-^ mice were left untreated (UN) or were treated with ionomycin (ION) or monosodium urate (MSU) crystals, then fixed, incubated with ACPA+ rheumatoid arthritis sera, permeabilized, and stained with DAPI (blue) and anti-human IgG antibody (pink). **(A)** Representative images at 400x. Graphs depict the means and individual values for the percentage of macrophages that are total METs **(B)**, ACPA-bound METs **(C)**, METs not bound by ACPAs **(D)**, and ACPA-bound non-MET macrophages **(E)**. For all panels: PAD2 ^+/+^ and PAD2^-/-^ were compared by paired t-test (*p<0.05, **p<0.01) and n=6.

Then, we determined if PAD4 or PAD2 is required to generate murine METs or activated macrophages that can be bound by ACPAs. We repeated the above ACPA experiments using wild-type, PAD4^-/-^, and PAD2^-/-^ mice. We found that in the absence of PAD4 (**Figure 5**) or PAD2 (**Figure 6**), there was a dramatic reduction in ACPA-bound METs and a lesser reduction in ACPA-bound non-MET macrophages. Interestingly, METs that were not ACPA-bound also were reduced in the absence of PAD2 or PAD4, potentially highlighting overall reduced binding by ACPA+ sera as compared to the anti-citH4 antibodies. Of note, we also performed the same experiment using each ACPA+ serum sample independently with overall similar results and some differences in staining patterns (**Supplementary Figures 4**, **5**). Taken together, our findings suggest that activated murine macrophages and METs have citrullinated proteins that are ACPA targets, and the generation of those citrullinated proteins is dependent upon both PAD4 and PAD2.

**Figure 7:**
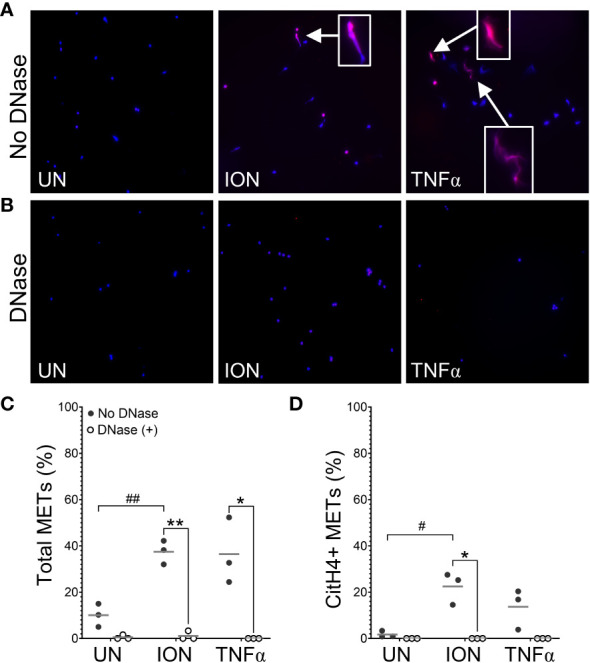
Human macrophages produce citrullinated histone-containing METs. Human peripheral blood-derived M1 macrophages were left untreated (UN) or were treated with ionomycin (ION) or tumor necrosis factor alpha (TNFα) followed by treatment with no DNase or DNase, then fixation and permeabilization, staining with DAPI (blue) and anti-citrullinated histone H4 (citH4) antibody (pink), imaging, and quantification of MET-like structures. Representative images at 200x of no DNase **(A)** or DNase **(B)** treated human macrophages. Arrows indicate citH4+ MET-like structures and insets are enlarged 3x. Graphs depict the means and individual values for the percent of macrophages that formed total **(C)** or citH4+ **(D)** MET-like structures for each stimulant compared to untreated for the non-DNase treated samples (#p<0.05, ##p<0.01) as well as for DNase compared to no DNase for each stimulant (*p<0.05, **p<0.01) by paired t-test (n=3).

**Figure 8:**
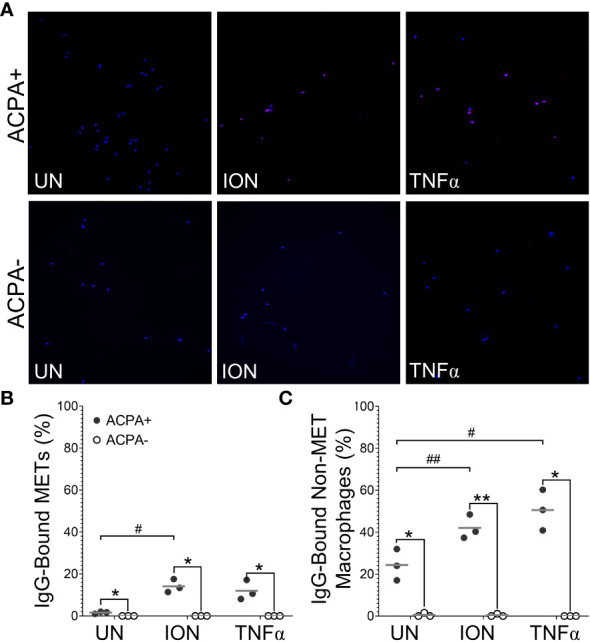
Anti-citrullinated protein antibodies (ACPAs) bind to human METs and activated non-MET macrophages. Untreated (UN), ionomycin (ION)-treated or tumor necrosis factor alpha (TNFα)-treated human M1 macrophages were fixed, incubated with sera from ACPA+ or ACPA- rheumatoid arthritis subjects, then permeabilized and stained with DAPI (blue) and anti-human IgG antibody (pink). **(A)** Representative images at 200x. Graphs depict the means and individual values for the percentage of macrophages that were IgG-bound METs **(B)** or IgG-bound non-MET macrophages **(C)** for each stimulant compared to the untreated control for ACPA+ sera (#p<0.05, ##p<0.01) and for ACPA+ versus ACPA- for each condition (*p<0.05, **p<0.01) by paired t-test (n=3).

Finally, to provide additional human connection, we evaluated the ability of human M1 macrophages to generate ACPA targets. First, we evaluated the capacity of ionomycin, MSU, and TNFα to generate human METs (**Figure 7**). We found that three concentrations of MSU led to a loss of almost all human macrophages from the coverslips. However, ionomycin induced citH4+ MET-like structures that were lost with DNase, indicating true citH4+ METs. TNFα gave similar citH4+ MET results that did not achieve statistical significance. We then evaluated the ability of IgG from human ACPA+ RA sera to bind to human macrophages and METs. As shown in **Figure 8**, IgG from ACPA+ sera bound ionomycin-induced human METs and non-MET macrophages more than IgG from ACPA- RA sera with similar results for TNFα. Taken together, these experiments provide the first evidence that human activated macrophages and METs generate citrullinated antigens bound by human RA ACPAs.

## Discussion

In this study, we report that, in response to ionomycin and MSU, murine macrophages generate citrullinated extracellular traps in a PAD4- and PAD2-dependent manner. We also show that ACPA+ sera can bind to murine and human METs, suggesting that METs can provide ACPA targets dependent upon PAD2 and PAD4.

Compared to NETs, the study of METs is only beginning. Our study investigated the ability of several stimuli to induce citH4+ and citH4- METs in thioglycolate-induced murine peritoneal macrophages (**Figure 1**). Although no murine model perfectly recapitulates human disease, stimulated murine peritoneal macrophages have a similar inflammatory phenotype as RA macrophages, allowing them to serve as a reasonable model of human pre-RA and RA macrophages (28, 29). In these cells, we demonstrate, for what we believe is the first time, that ionomycin, MSU, and PAF induce citH4+ METs. We also demonstrate for the first time that ionomycin induces human citH4+ METs, although a different calcium ionophore was previously shown to induce METs detected by DNA staining (30). In contrast, three concentrations of MSU led to a loss of human macrophages in general, perhaps consistent with MSU being unable to induce extracellular traps in human mononuclear cells (31).

We did not observe MET production in response to some stimuli, such as PMA or LPS, consistent with previous findings for murine peritoneal macrophages (16). This finding is in contrast to the ability of LPS and PMA to induce METs in murine splenic macrophages (19), suggesting that tissue of origin may influence METosis. Interestingly, human monocyte-derived macrophages generate METs in response to PMA (30), consistent with our previous observation that PMA induces NETs in human, but not murine, neutrophils (10). We also found that heat-killed *Candida* induced nuclear enlargement, but not extracellular traps. Previous studies report that murine macrophages can form METs in response to live *Candida* (16, 32), so perhaps live *Candida* induces more METosis than heat-killed *Candida* in murine macrophages. Finally, we found that TNFα induced primarily citH4- METs in mice. This systematic evaluation of citrullination and confirmation of extracellular structures with DNase provides new insights into murine MET activators. Similar studies are needed for human METs, given reported differences for human and murine NETs and different methodologies (10).

Using both human and murine METs, we also demonstrate that METs are a potential source of citrullinated autoantigens bound by ACPAs, similar to NETs (11, 12), although the relative contributions of METs and NETs in the initiation and propagation of RA inflammation are unknown. Others have demonstrated that human and murine METs are citrullinated (15, 19), but, to the best of our knowledge, this study is the first to demonstrate that human RA ACPAs bind to murine and human METs. IgG from ACPA+ human RA sera bound murine METs due either to conserved epitopes between mice and humans or due to the previously demonstrated multi-reactivity of ACPAs related to the recognition of very short motifs (33, 34). A caveat to our findings is the use of ACPA+ sera, not purified ACPAs; thus, we cannot exclude the possibility that a non-ACPA IgG, such as rheumatoid factor or an anti-homocitrullinated protein antibody, in the ACPA+ sera bound to the murine METs. However, multi-reactive ACPAs have rheumatoid factor activity (35) and can bind to homocitrullinated antigens (33), so eliminating all multi-reactivity while keeping ACPAs may not be possible. Nonetheless, IgG binding occurred using ACPA+ and not ACPA- RA sera, indicating that RA autoantibodies can bind METs. Thus, METs are a target of immune tolerance loss in RA, likely due to the presence of citrullinated proteins given the dependence on PAD2 and PAD4. Future studies are needed to evaluate the binding of purified ACPAs to human METs as well as to address the relative contributions of METs and NETs in the initiation and propagation of RA inflammation.

Additionally, we show that both PAD2 and PAD4 are required for the production of citrullinated, ACPA-bound murine METs. For PAD4, this finding is similar to the requirement for PAD4 in citrullinated NETs (10). However, the finding that PAD2 is required for citrullinated METs (**Figures 3**, **6**) is, to the best of our knowledge, the first demonstration that PAD2 is required for any form of ETosis. Using knockout mice, we previously found that PAD2 was dispensable for NET formation (5, 10). Also, PADs, and PAD2 in particular, were found to be dispensable for human monocyte-derived macrophage ETosis based on inhibitor and PAD2 siRNA experiments (30). It is not clear if the discrepancy with that study is due to our complete genetic knockout of PAD2 versus incomplete PAD inhibition by Cl-amidine or siRNA, our use of peritoneal macrophages versus peripheral blood monocyte-derived macrophages, differences between mice and humans, or our quantification of METs versus representative images. A requirement for PAD2 in METosis provides a potential mechanism for the observation that PAD2 is required for the citrullination seen in murine arthritis (5) and the close correlation of synovial fluid PAD2 levels with synovial fluid PAD activity and RA disease activity (36). It will be important for future studies to thoroughly evaluate a role for PADs in human METs using a variety of methods.

A caveat to our citrullination findings is that we quantified citrullinated METs using an antibody that detects citrullination of Arg3 in histone H4, a citrulline that may specifically depend upon PAD4 (37). The use of this antibody may explain the complete loss of citH4+ METs in PAD4^-/-^ macrophages versus the partial loss in PAD2^-/-^ macrophages. We previously used F95 as a pan anti-citrulline antibody (10), but recent lots of F95 did not detect citrullination in our hands (data not shown). However, similar results were seen with F95 and anti-citrullinated histone H4 antibody for NETs (10). Moreover, the ACPA+ serum experiments provide similar results to the anti-citrullinated histone experiments.

Finally, we quantified citH4+ and ACPA-bound non-METs in addition to METs, providing some interesting insights. For example, ACPA-bound non-MET macrophages are not sensitive to DNase, suggesting an intact nuclear membrane (protecting DNA from DNase) and a permeable cell membrane (allowing ACPA entry to the cytoplasm). These findings could be due to the pore-forming capabilities of ionomycin and MSU (38–40) or a mechanism of ETosis in which cell membrane permeabilization is an early event as opposed to the early loss of nuclear membrane integrity in NETosis (41). Either way, non-MET macrophages might also provide some citrullinated antigen with the relative contributions of METs and activated non-MET macrophages unknown. Also, non-MET macrophages with citrullinated histone H4 are lost in the absence of PAD4, whereas they are minimally reduced in the absence of PAD2. In contrast, ACPA-bound non-MET macrophages were only minimally reduced in the absence of PAD2 or PAD4. Since the non-MET macrophages may be early or incomplete METs, PAD4 may be required early in METosis to citrullinate histone H4, whereas PAD2 is more dispensable early for histone H4 citrullination. Also, whereas PAD4 appears to be absolutely required for histone H4 citrullination, PAD2 and PAD4 can partially compensate for each other in the citrullination of other targets in early METosis. In contrast, PAD2 and PAD4 are each required independently for full METosis.

In sum, our work demonstrates that both PAD2 and PAD4 are required for the production of citrullinated METs that are bound by ACPAs. Although further work is needed to fully evaluate PAD2 and PAD4 in human METs, our findings provide a new potential source of citrullinated antigens bound by ACPAs in RA and demonstrate the importance of both PAD2 and PAD4 in the generation of those antigens.

## Data availability statement

The original contributions presented in the study are included in the article/**Supplementary Material**. Further inquiries can be directed to the corresponding author.

## Ethics statement

The studies involving humans were approved by University of Wisconsin Institutional Review Board. The studies were conducted in accordance with the local legislation and institutional requirements. The participants provided their written informed consent to participate in this study. The animal study was approved by University of Wisconsin Institutional Animal Care and Use Committee. The study was conducted in accordance with the local legislation and institutional requirements.

## Author contributions

SB generated and analyzed data, prepared figures, and wrote the first draft of the manuscript. CH generated and analyzed data. MS conceptualized and oversaw the research. All authors edited the manuscript and approved the final version.
