# Supplementary material for: Macrophage extracellular traps require peptidylarginine deiminase 2 and 4 and are a source of citrullinated antigens bound by rheumatoid arthritis autoantibodies
Source: Front Immunol. 2024 Feb 27;15:1167362. doi: 10.3389/fimmu.2024.1167362 (PMC10927735; doi:10.3389/fimmu.2024.1167362)
Supplement: Supplementary file 1 [file Presentation_1.pdf]

Supplementary Material

Macrophage Extracellular Traps Require Peptidylarginine Deiminase 2 And 4 and are a Source of Citrullinated Antigens Bound by Rheumatoid Arthritis Autoantibodies

S. Janna Bashar, Caitlyn L. Holmes, Miriam A. Shelef *

*** Correspondence:** Miriam A. Shelef, MD, PhD: mshelef@medicine.wisc.edu

1 Supplementary Figures

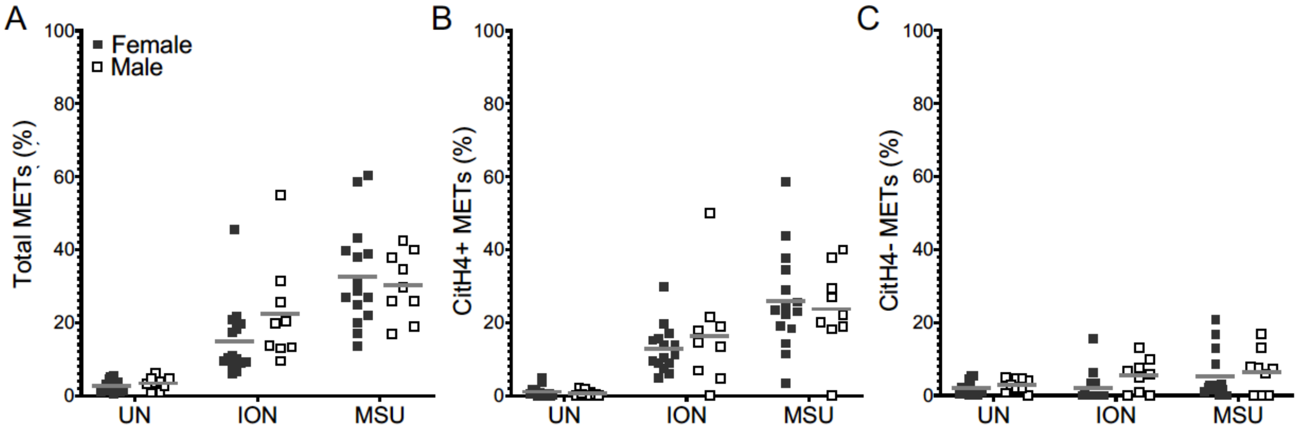

Supplementary Figure 1. Similar MET formation in female and male mice. Peritoneal macrophages from male (n=9) and female (n=15) mice were left untreated (UN) or were treated with ionomycin (ION) or monosodium urate (MSU) crystals, stained with DAPI and anti-citrullinated histone H4 (citH4) antibody, and METs were quantified. Graphs show means and individual values for the percentage of total METs (A), citH4+ METs (B), and citH4- METs (C) for each condition. For all panels, no significant difference was found between male and female mice by unpaired t-test.

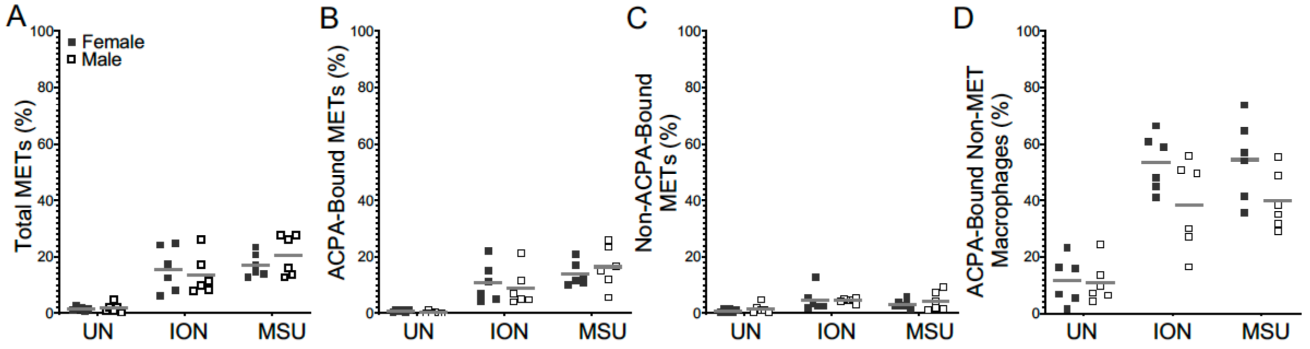

Supplementary Figure 2. Similar ACPA-bound METs and activated macrophages in female and male mice. Peritoneal macrophages from male (n=6) and female (n=6) mice were left untreated or were treated with ionomycin (ION) or monosodium urate (MSU) crystals, then incubated with ACPA+ RA sera, permeabilized, and stained with DAPI and anti-human IgG antibody, followed by MET quantification. Graphs show means and individual values for the percentage of total METs (A), ACPA-bound METs (B), METs not bound by ACPAs (C), and ACPA-bound non-MET macrophages (D) for each condition. For all panels, no significant difference was found between male and female mice by unpaired t-test.

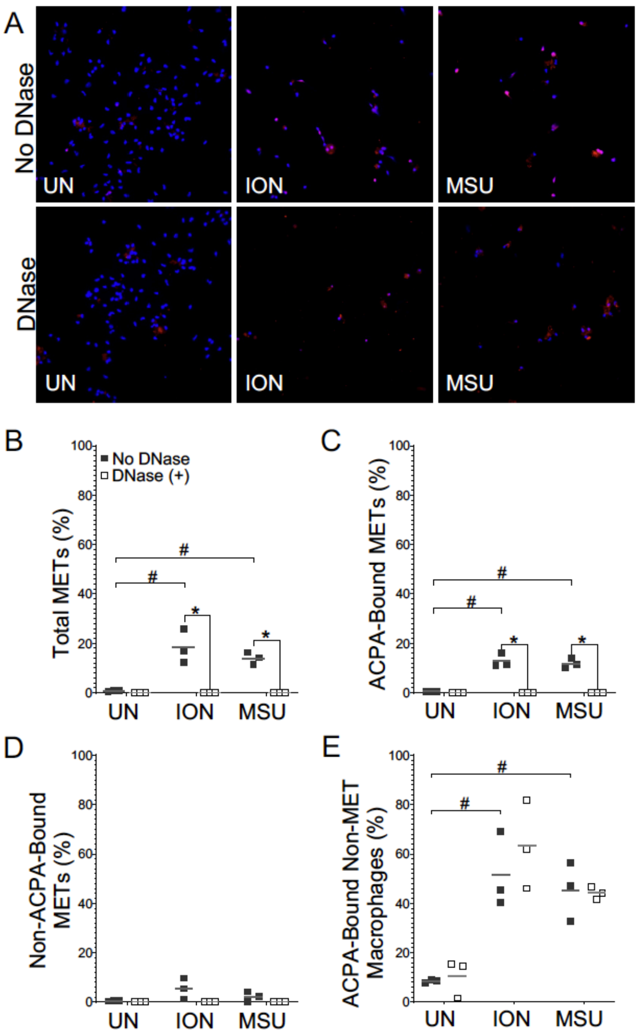

Supplementary Figure 3. Anti-citrullinated protein antibody (ACPA)-bound non-MET macrophages are unaffected by DNase. . Untreated (UN), ionomycin (ION)-treated or monosodium urate (MSU) crystal-treated murine macrophages were incubated with no DNase or DNase, fixed, incubated with sera from ACPA+ or ACPA- rheumatoid arthritis subjects, permeabilized, then stained with DAPI (blue) and anti-human IgG antibody (pink). (A) Representative images at 400x. Graphs depict the means and individual values for the percentage of macrophages that are total METs (B), ACPA-bound METs (C), METs not bound by ACPAs (D), or ACPA-bound non-MET macrophages (E). Cellular structures for each stimulant were compared to untreated for the non-DNase treated samples (#$p<0.05$) as well as for DNase compared to no DNase for each stimulant (*$p<0.05$) by paired t-test (n=3).

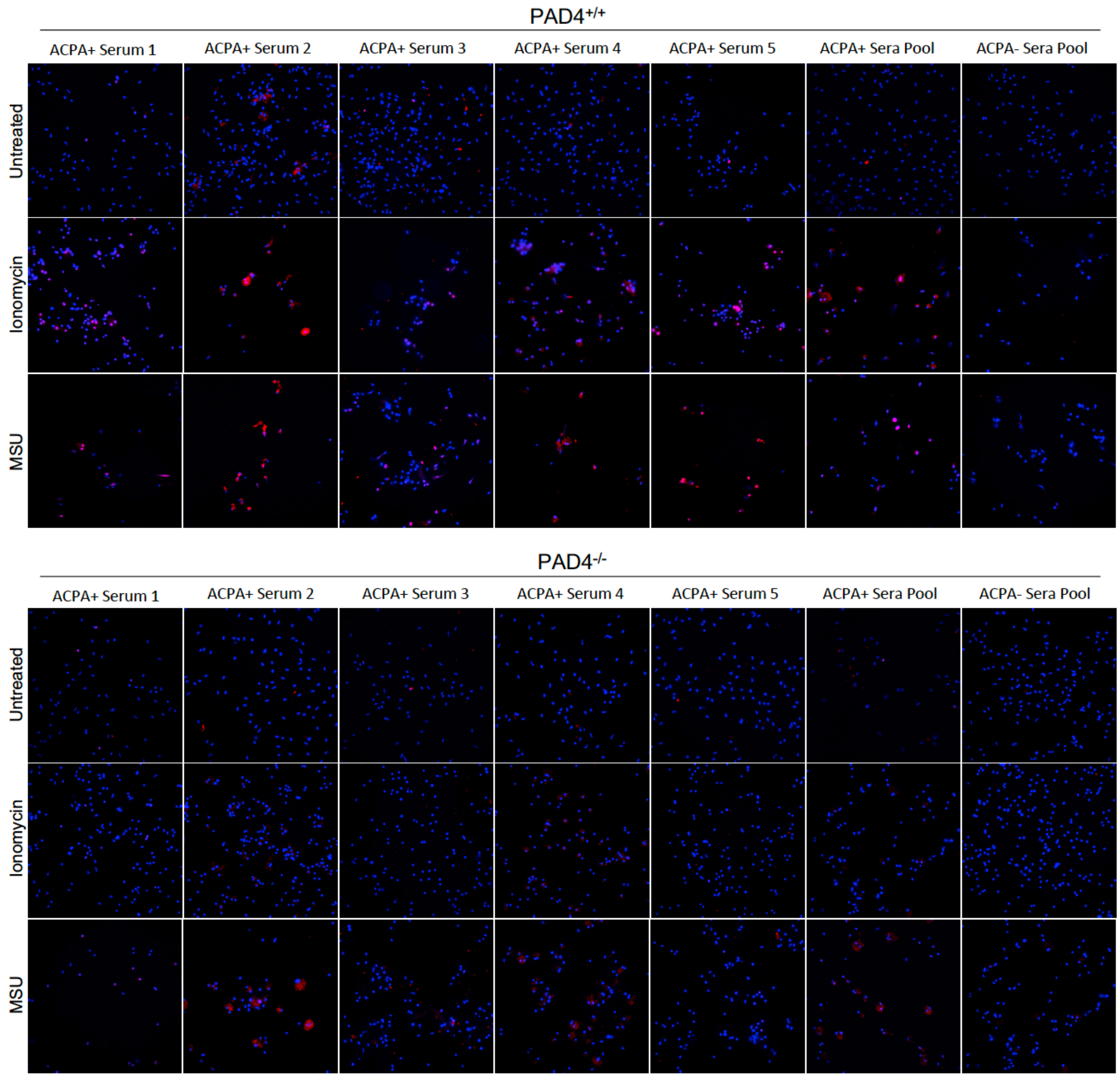

Supplementary Figure 4. Similar results for individual ACPA+ serum samples as pooled ACPA+ sera in the presence and absence of PAD4. Peritoneal macrophages from $PAD4^{+/+}$ and $PAD4^{-/-}$ mice were left untreated (UN) or were treated with ionomycin (ION) or monosodium urate (MSU) crystals then fixed, incubated with sera from individual ACPA+ rheumatoid arthritis subjects, pooled sera from the five ACPA+ rheumatoid arthritis subjects, or pooled sera from five ACPA- rheumatoid arthritis subjects. Cells were then permeabilized, stained with DAPI (blue) and anti-human IgG antibody (pink), and imaged at 400x. Representative images for one experiment are shown.

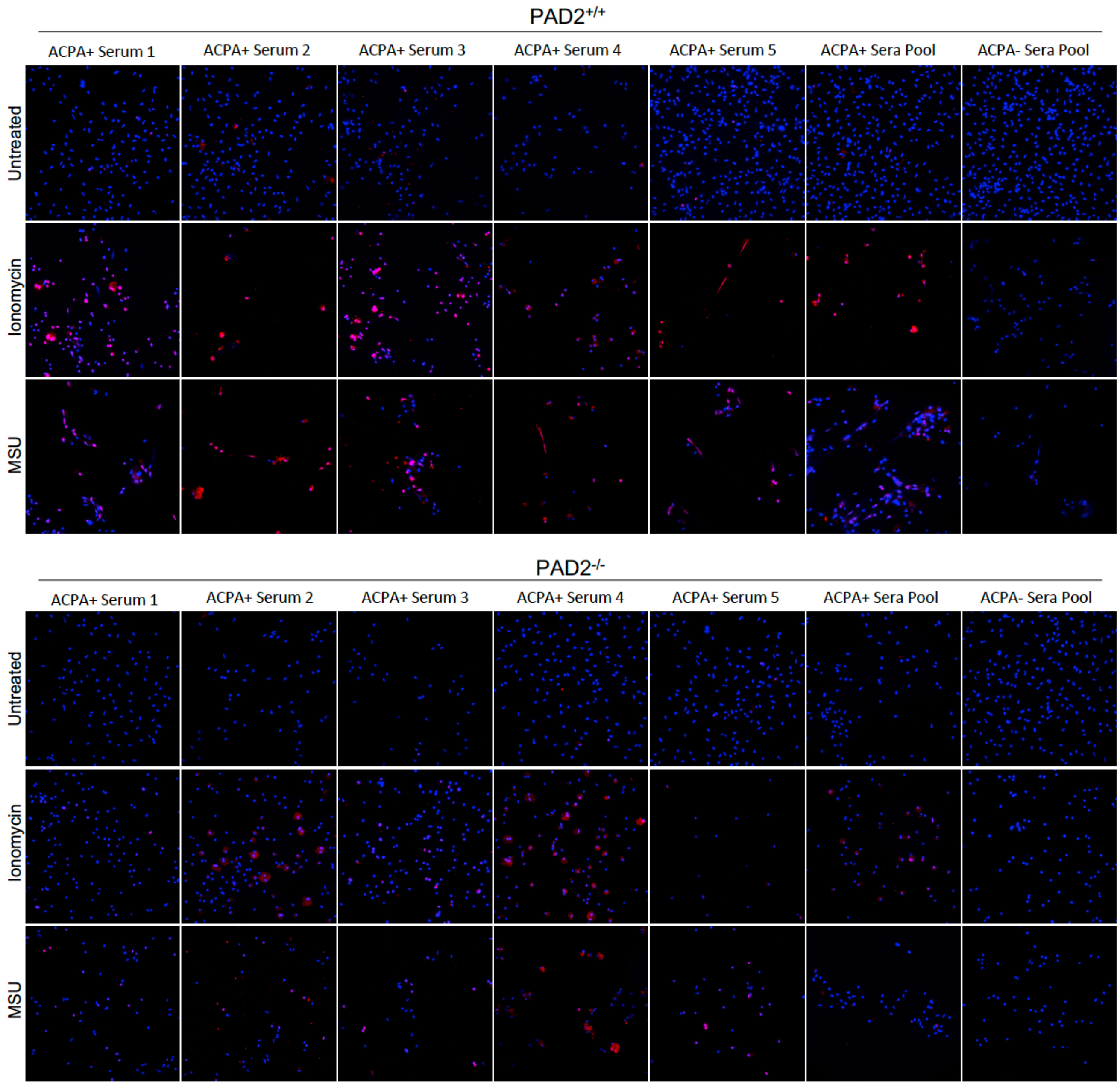

Supplementary Figure 5. Similar results for individual ACPA+ serum samples as pooled ACPA+ sera in the presence and the absence of PAD2. Peritoneal macrophages from PAD2$^{+/+}$ and PAD2$^{-/-}$ mice were left untreated (UN) or were treated with ionomycin (ION) or monosodium urate (MSU) crystals then fixed, incubated with sera from individual ACPA+ rheumatoid arthritis subjects, pooled sera from the five ACPA+ rheumatoid arthritis subjects, or pooled sera from five ACPA- rheumatoid arthritis subjects. Cells were then permeabilized, stained with DAPI (blue) and anti-human IgG antibody (pink), and imaged at 400x. Representative images for one experiment are shown.
